# Supplementary material for: Synthesis and Characterization of Bulky Substituted Hemihexaphyrazines Bearing 2,6-Diisopropylphenoxy Groups
Source: Molecules. 2023 Jul 29;28(15):5740. doi: 10.3390/molecules28155740 (PMC10421089; doi:10.3390/molecules28155740)
Supplement: Supplementary file 1 [file molecules-28-05740-s001.zip › molecules-2438130-supplementary.pdf]

Supplementary Materials

Synthesis and characterization of bulky substituted hemihexaphyrazines bearing 2,6-diisopropylphenoxy groups

Evgenii N. Ivanov [1,2], Verónica Almeida-Marrero [3], Oskar I. Koifman [1,2], Viktor V. Aleksandriiskii [1,2], Tomas Torres, [3, 4, 5, *] and Mikhail K. Islyaikin [1, 2, *]

1 IRLoN, Research Institute of Macroheterocycles, Ivanovo State University of Chemistry and Technology, 7, Sheremetievskiy Ave., 153000 Ivanovo, Russia; ivanov.isuct@gmail.com (E.N.I.); oik@isuct.ru (O.I.K.), nmr@isuct.ru (V.V.A.)
2 G. A. Krestov Institute of Solution Chemistry of the Russian Academy of Sciences, 1 Akademicheskaya Str., 153045 Ivanovo, Russian Federation
3 Department of Organic Chemistry, Autonoma University of Madrid, Cantoblanco, 28049 Madrid, Spain; tomas.torres@uam.es
4 Institute for Advanced Research in Chemical Sciences (IAdChem), Autonoma University of Madrid, 28049 Madrid, Spain
5 Instituto Madrileño de Estudios Avanzados (IMDEA)-Nanociencia, c/ Faraday 9, Cantoblanco, 28049 Madrid, Spain
* Correspondence: tomas.torres@uam.es (T.T.); mkimailster@gmail.com; Tel.: +79106847175 (M.K.I.)

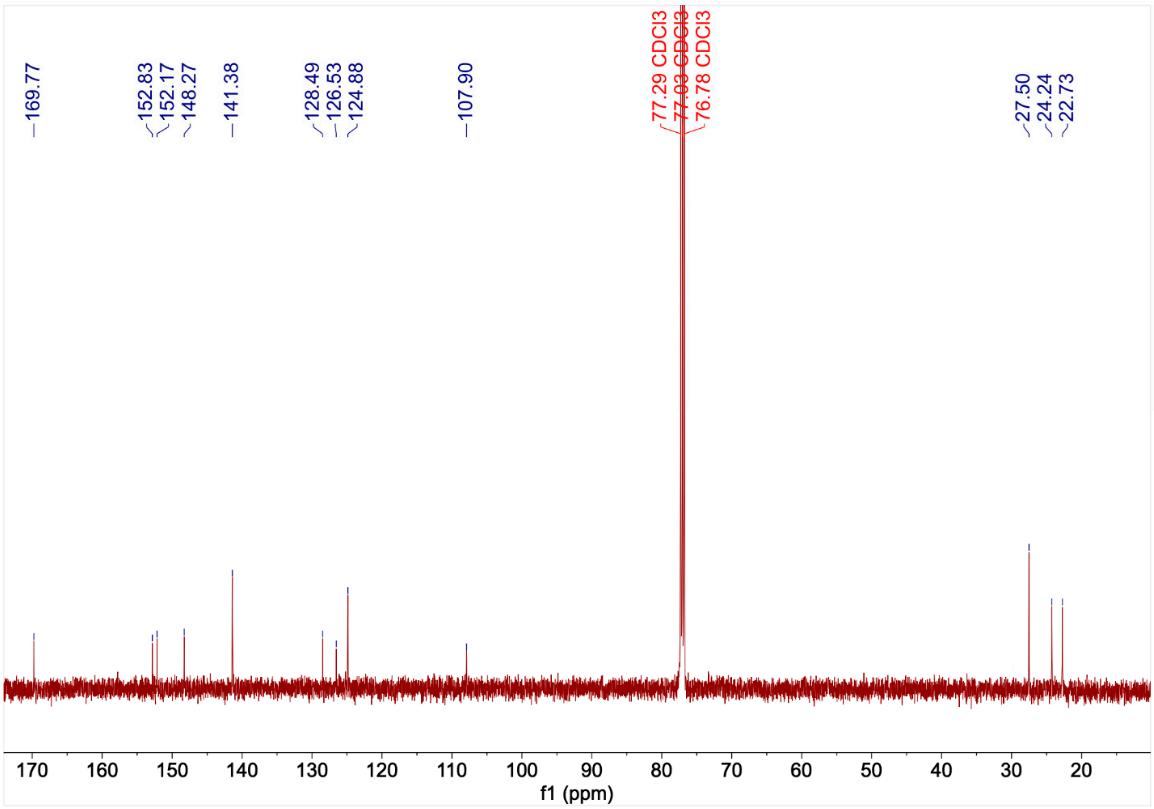

Figure S1: ^{13}C-NMR spectrum of **4** in $CDCl_3$

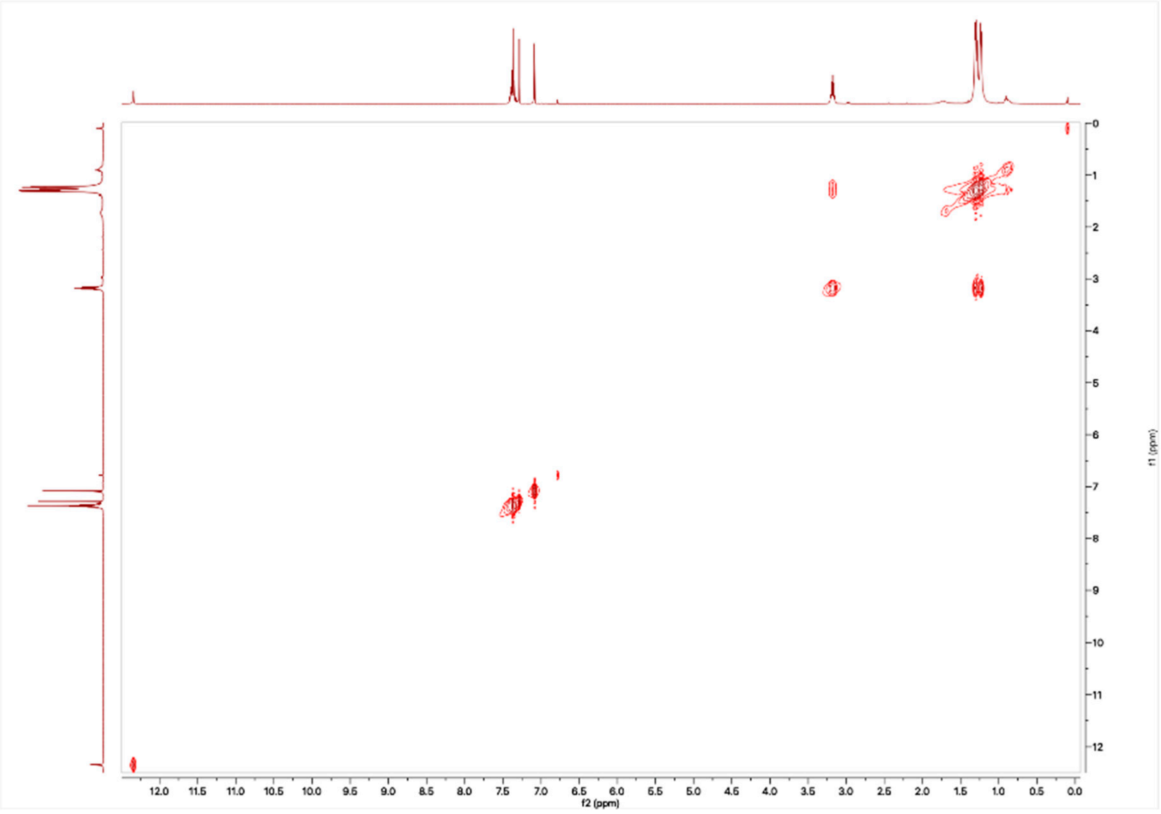

Figure S2: COSY ^{1}H-^{1}H NMR spectrum of **4** in $CDCl_3$

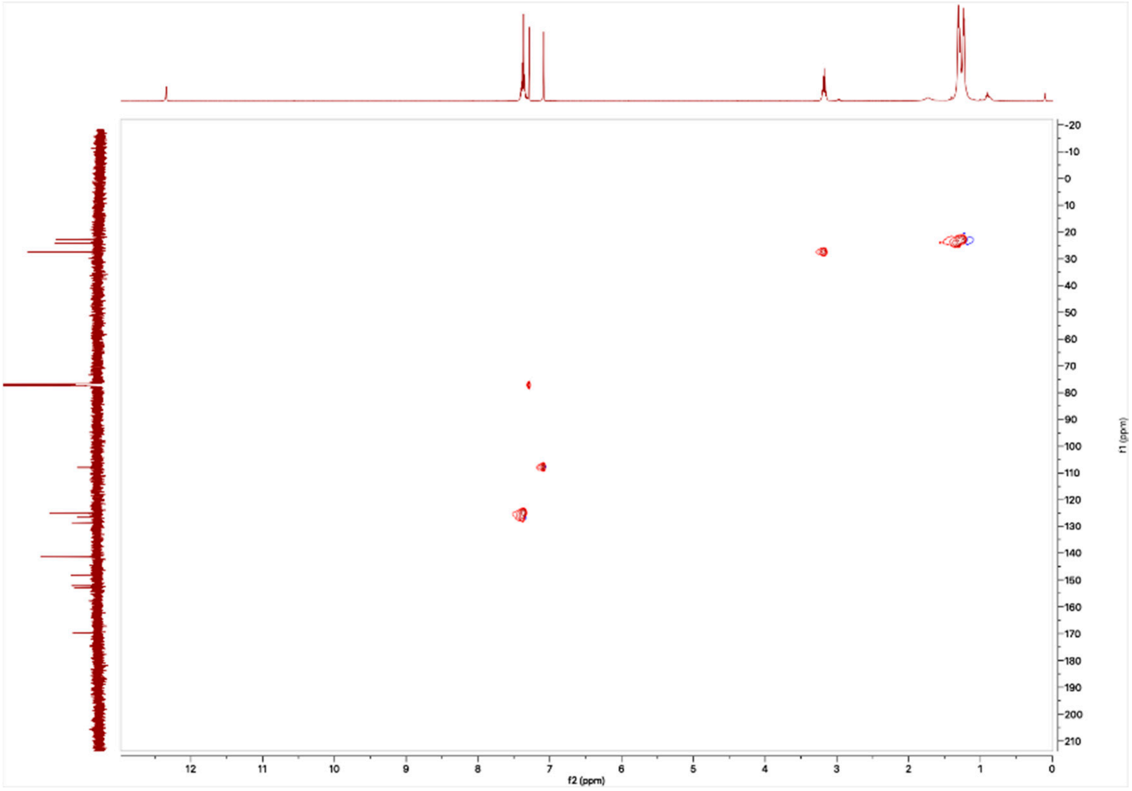

Figure S3: HSQC ^1H-^{13}C NMR spectrum of **4** in $CDCl_3$

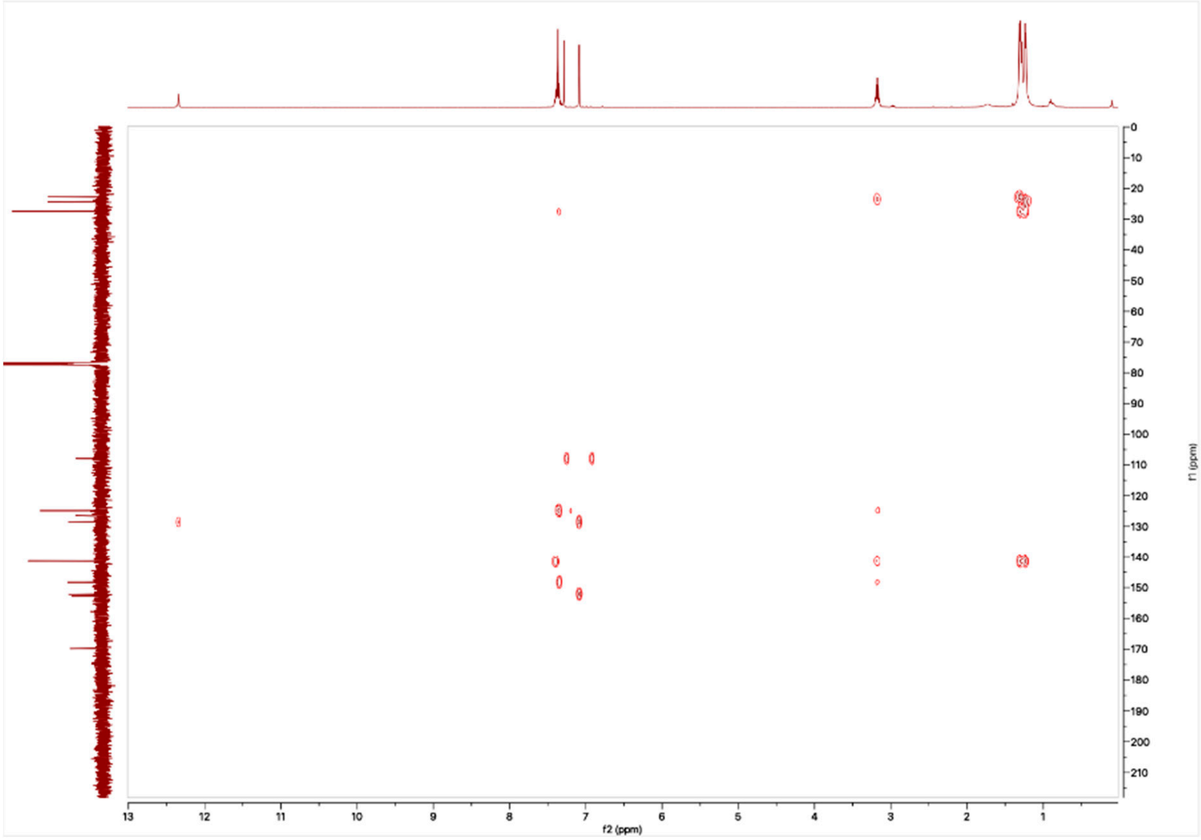

Figure S4: HMBC ^1H-^{13}C NMR spectrum of **4** in $CDCl_3$

Table S1. Experimental (δ_{exp}) and computational (δ_{calc}) (GIAO) chemical shifts **(4)** ^{13}C и ^{1}H

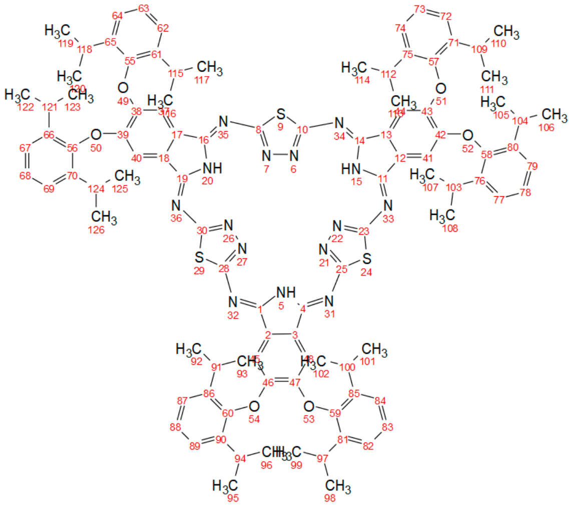

C	δ_{exp}	HSQC	HMBC				δ_{calc}
8,10	169.77						171.35
111,114	152.82		12.34				151.84
12,13	128.49		12.34	7.08			129.03
41	107.9	7.08					119.04
42,43	152.17			7.08			152.20
44	107.9	7.08					119.04
57	148.27		7.35				151.37
71,75	141.38		7.38	3.2	1.31	1.24	138.13
72	124.88	7.35	7.38				129.78
73	126.63	7.38					122.53
109,112	27.5	3.18					39.20
110,113	24.24	1.31		3.2	1.24		24.72
111,114	22.73	1.24			1.31		21.41
						MAE*	1.696
						R^2	0.992

H	δ_{exp}	δ_{calc}
15	12.34	12.03
41,44	7.08	8.02
72,74	7.38	7.31
73	7.35	7.32
109,112	3.18	3.07
110,113	1.31	1.13
111,114	1.24	0.94
	MAE	0.070
	R^2	0.984

$$* MAE = \frac{\sum_{i=1}^{n} \left| \delta_{\exp} - \delta_{calc} \right|_i}{n}$$

R^2 - linear correlation coefficient

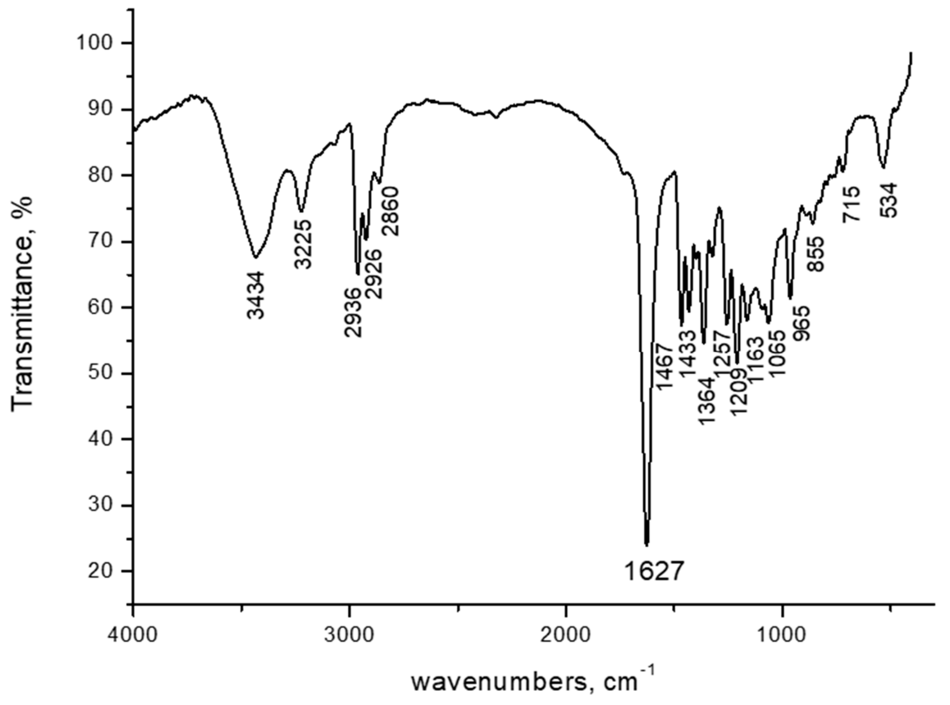

Figure S5: IR spectrum of **3** in palette with KBr

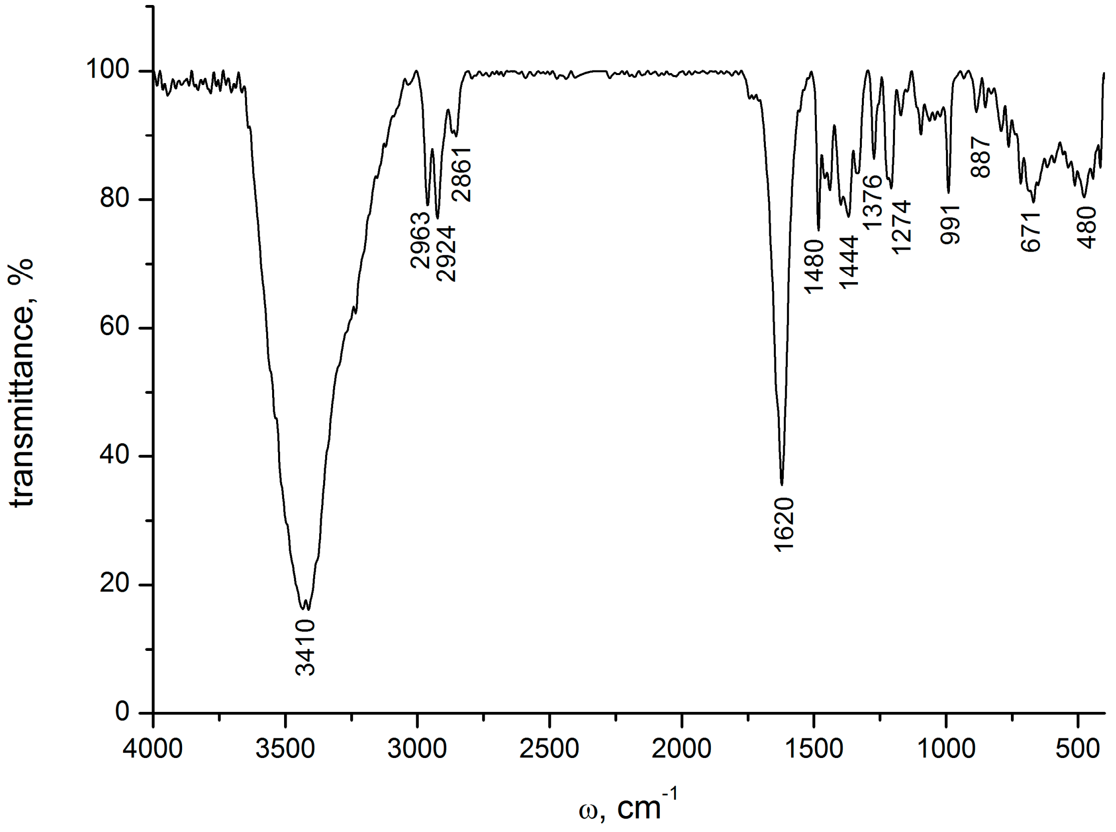

Figure S6: IR spectrum of **4** in palette with KBr

Table S2. a) Geometry parameters (Å) and E_{tot} (a.u.) of **4** optimized at DFT/CAM-B3LYP/6-31G(d,p) method

N	0.605448000	-2.673510000	-0.002625000
C	-1.560122000	-4.515174000	-0.000713000
C	-3.482671000	-3.267893000	0.002053000
N	-2.098696000	-3.239990000	0.000266000
C	-3.865660000	-4.687591000	-0.000951000
C	-2.700530000	-5.443600000	0.002808000
N	-0.331588000	-4.888084000	-0.001851000
N	-4.324003000	-2.298088000	0.003052000
C	-3.126561000	3.609806000	0.005296000
C	-1.085203000	4.651364000	0.000148000
N	-1.752902000	3.438811000	0.002962000
C	-2.123373000	5.692824000	-0.002850000
C	-3.360524000	5.061538000	0.006341000
N	0.175319000	4.895101000	-0.001833000
N	-4.063621000	2.732153000	0.008185000
C	4.692525000	0.903613000	0.003210000
C	4.574211000	-1.385062000	-0.004776000
N	3.857768000	-0.200616000	-0.000378000
C	6.066816000	0.380532000	0.005047000
C	5.995160000	-1.006481000	-0.006090000
N	4.401012000	2.153946000	0.006483000
N	4.155118000	-2.598624000	-0.008892000
C	-3.911468000	-0.993050000	0.004555000
C	-3.788051000	1.391475000	0.006622000
S	-5.116197000	0.264759000	0.007252000
N	-2.684106000	-0.539344000	0.003531000
C	1.099131000	3.885189000	0.000688000
S	2.790823000	4.299376000	0.002789000
N	2.014590000	1.858223000	0.002948000
C	3.102232000	2.585760000	0.004112000
C	0.691785000	-3.979198000	-0.003905000
S	2.331600000	-4.566122000	-0.007489000
N	1.812026000	-2.057833000	-0.004304000
C	2.818636000	-2.893911000	-0.006772000
H	-1.542936000	-2.383517000	-0.000177000
H	-1.288914000	2.529342000	0.003136000
H	2.838142000	-0.147909000	-0.000131000
C	-2.757082000	-6.818395000	0.049570000
C	-3.998443000	-7.464640000	0.088229000
C	-5.182606000	-6.696295000	-0.084705000
C	-5.098168000	-5.299440000	-0.046605000
C	-2.037477000	7.065940000	-0.053333000
C	-3.205335000	7.837233000	-0.090493000
C	-4.462045000	7.196175000	0.088660000
C	-4.522789000	5.797947000	0.054255000
C	7.141683000	-1.767089000	-0.055988000
C	8.393516000	-1.141135000	-0.090375000
C	8.466132000	0.267589000	0.090639000
C	7.285457000	1.019047000	0.055526000
N	0.878182000	2.595433000	0.001271000

N	-2.614096000	0.813439000	0.004548000
H	-1.857481000	-7.419748000	0.086790000
H	-6.013856000	-4.722824000	-0.082889000
H	-1.080605000	7.570892000	-0.094903000
H	-5.493201000	5.319468000	0.094800000
H	7.100768000	-2.848200000	-0.098677000
H	7.356015000	2.098631000	0.098306000
O	9.588851000	1.039242000	0.189961000
C	10.673171000	0.835304000	1.037623000
C	11.931544000	1.035754000	0.447719000
C	10.530892000	0.609177000	2.424789000
C	13.056945000	0.744510000	1.221692000
C	11.701772000	0.342779000	3.138740000
H	14.036845000	0.841785000	0.763955000
H	11.619825000	0.141786000	4.202425000
C	12.953047000	0.359264000	2.545076000
H	13.840433000	0.131647000	3.126240000
C	9.272191000	0.800819000	3.280400000
H	9.669923000	0.819203000	4.300385000
C	8.226533000	-0.322004000	3.294039000
C	8.639082000	2.180695000	3.069313000
H	9.381170000	2.971632000	3.205629000
H	8.211493000	2.292739000	2.074141000
H	7.836209000	2.339250000	3.795197000
H	7.621606000	-0.359666000	2.390602000
H	7.544233000	-0.158598000	4.133765000
H	8.693513000	-1.299967000	3.432645000
C	12.224753000	1.661542000	-0.921021000
H	12.976756000	1.013288000	-1.386180000
C	12.873474000	3.040245000	-0.695706000
C	11.092749000	1.828661000	-1.934463000
H	10.581952000	0.892947000	-2.140985000
H	10.353263000	2.557814000	-1.598264000
H	11.522088000	2.193114000	-2.873282000
H	12.161870000	3.722371000	-0.220117000
H	13.170693000	3.479074000	-1.652828000
H	13.758615000	2.986858000	-0.058656000
O	9.431364000	-2.023822000	-0.188946000
C	10.530334000	-1.933130000	-1.037393000
C	11.761514000	-2.262741000	-0.448044000
C	10.411632000	-1.693510000	-2.424509000
C	12.910643000	-2.089461000	-1.222514000
C	11.603518000	-1.549382000	-3.138875000
H	13.875416000	-2.287848000	-0.765298000
H	11.542332000	-1.341003000	-4.202534000
C	12.846628000	-1.695205000	-2.545793000
H	13.752549000	-1.560507000	-3.127324000
C	9.139791000	-1.755314000	-3.279953000
H	9.533488000	-1.815138000	-4.299909000
C	8.214890000	-0.531082000	-3.294453000
C	8.368345000	-3.062710000	-3.067986000
H	9.025515000	-3.925774000	-3.202550000
H	7.930485000	-3.129083000	-2.073191000

H	7.554159000	-3.139030000	-3.794585000
H	7.617110000	-0.430832000	-2.391056000
H	7.519346000	-0.624294000	-4.134044000
H	8.779720000	0.393687000	-3.433882000
C	11.989189000	-2.916037000	0.920332000
H	12.804127000	-2.348893000	1.385425000
C	12.492449000	-4.354077000	0.694133000
C	10.846502000	-2.966291000	1.934330000
H	10.435333000	-1.983032000	2.142239000
H	10.035419000	-3.614684000	1.597769000
H	11.236332000	-3.374279000	2.872476000
H	11.713933000	-4.959275000	0.219260000
H	12.744028000	-4.821370000	1.650863000
H	13.377684000	-4.391882000	0.056093000
O	-3.892206000	-8.822981000	0.183626000
C	-4.610160000	-9.661704000	1.030476000
C	-5.072631000	-10.847497000	0.437558000
C	-4.727208000	-9.430960000	2.419221000
C	-5.886171000	-11.677284000	1.212268000
C	-5.541980000	-10.312463000	3.133729000
H	-6.297559000	-12.571020000	0.752741000
H	-5.669026000	-10.145161000	4.198819000
C	-6.159759000	-11.399848000	2.538420000
H	-6.799522000	-12.055189000	3.120010000
C	-3.925421000	-8.441597000	3.274250000
H	-4.103569000	-8.798553000	4.293950000
C	-4.373100000	-6.974086000	3.295164000
C	-2.415294000	-8.584870000	3.054653000
H	-2.102118000	-9.623731000	3.187148000
H	-2.109487000	-8.269159000	2.058401000
H	-1.871648000	-7.971133000	3.778839000
H	-4.106067000	-6.428401000	2.392671000
H	-3.886678000	-6.468547000	4.134920000
H	-5.452951000	-6.888861000	3.438068000
C	-4.687927000	-11.408620000	-0.936552000
H	-5.629724000	-11.726947000	-1.398948000
C	-3.824698000	-12.666354000	-0.723633000
C	-3.976752000	-10.509991000	-1.948062000
H	-4.527560000	-9.595609000	-2.147133000
H	-2.972579000	-10.241571000	-1.614638000
H	-3.882895000	-11.059295000	-2.890395000
H	-2.874014000	-12.399819000	-0.251378000
H	-3.601192000	-13.138477000	-1.684948000
H	-4.314466000	-13.407384000	-0.088711000
O	-6.466338000	-7.153895000	-0.176445000
C	-6.944571000	-8.143741000	-1.029182000
C	-7.846317000	-9.045163000	-0.440705000
C	-6.683711000	-8.154599000	-2.417573000
C	-8.277893000	-10.121478000	-1.218836000
C	-7.161567000	-9.253796000	-3.135362000
H	-8.932950000	-10.857667000	-0.762608000
H	-6.955139000	-9.300320000	-4.200142000
C	-7.910381000	-10.257888000	-2.544332000

H	-8.251955000	-11.105720000	-3.128727000
C	-6.102403000	-7.019699000	-3.270565000
H	-6.354997000	-7.326543000	-4.290825000
C	-4.579625000	-6.832667000	-3.290103000
C	-6.847107000	-5.698092000	-3.051176000
H	-7.923344000	-5.833870000	-3.185875000
H	-6.684764000	-5.291089000	-2.054324000
H	-6.505720000	-4.951368000	-3.774032000
H	-4.189668000	-6.369112000	-2.386506000
H	-4.315022000	-6.180947000	-4.128326000
H	-4.063083000	-7.784428000	-3.435220000
C	-8.520268000	-8.920292000	0.930908000
H	-8.438628000	-9.912461000	1.390305000
C	-10.016980000	-8.630189000	0.711651000
C	-7.984832000	-7.913248000	1.948541000
H	-6.927458000	-8.053888000	2.151614000
H	-8.138346000	-6.884186000	1.618383000
H	-8.530059000	-8.050156000	2.887968000
H	-10.149809000	-7.650360000	0.242440000
H	-10.543951000	-8.617463000	1.670364000
H	-10.497414000	-9.372726000	0.071351000
O	-2.959041000	9.177013000	-0.190282000
C	-3.589396000	10.083179000	-1.037380000
C	-3.923088000	11.312637000	-0.446914000
C	-3.736116000	9.861374000	-2.424753000
C	-4.649320000	12.220115000	-1.221234000
C	-4.458184000	10.820541000	-3.138991000
H	-4.962989000	13.153462000	-0.763369000
H	-4.606820000	10.663992000	-4.202917000
C	-4.956609000	11.968272000	-2.545122000
H	-5.527315000	12.684784000	-3.126436000
C	-3.044916000	8.791742000	-3.279773000
H	-3.189138000	9.162570000	-4.299878000
C	-3.642352000	7.378513000	-3.294630000
C	-1.527212000	8.778051000	-3.066069000
H	-1.108711000	9.778840000	-3.200750000
H	-1.251774000	8.432858000	-2.070707000
H	-1.052912000	8.110936000	-3.791777000
H	-3.430717000	6.810839000	-2.391023000
H	-3.213412000	6.822730000	-4.133961000
H	-4.725619000	7.405301000	-3.434439000
C	-3.474673000	11.835695000	0.922941000
H	-4.375526000	12.253108000	1.388039000
C	-2.485295000	12.994985000	0.700348000
C	-2.856792000	10.871616000	1.935509000
H	-3.499560000	10.021010000	2.141458000
H	-1.888020000	10.497754000	1.598990000
H	-2.701126000	11.412056000	2.874781000
H	-1.569932000	12.628039000	0.225666000
H	-2.209220000	13.445449000	1.658392000
H	-2.897517000	13.780355000	0.063652000
O	-5.691408000	7.784070000	0.183940000
C	-6.060233000	8.821700000	1.034367000

C	-6.865258000	9.810317000	0.445704000
C	-5.794123000	8.810049000	2.421724000
C	-7.178647000	10.928708000	1.221086000
C	-6.151302000	9.955815000	3.136908000
H	-7.754904000	11.727783000	0.764349000
H	-5.936799000	9.984292000	4.200734000
C	-6.793304000	11.030653000	2.544624000
H	-7.041838000	11.911655000	3.126921000
C	-5.330907000	7.623650000	3.276970000
H	-5.547466000	7.958100000	4.296835000
C	-3.835486000	7.280610000	3.293097000
C	-6.208557000	6.385249000	3.063963000
H	-7.264933000	6.632292000	3.197692000
H	-6.089394000	5.958677000	2.069215000
H	-5.946126000	5.610745000	3.790570000
H	-3.497454000	6.779182000	2.388803000
H	-3.637426000	6.605293000	4.131013000
H	-3.223303000	8.174133000	3.436515000
C	-7.554100000	9.751342000	-0.922954000
H	-7.372058000	10.728080000	-1.386593000
C	-9.071918000	9.618617000	-0.697008000
C	-7.130048000	8.690561000	-1.938489000
H	-6.064593000	8.720110000	-2.146018000
H	-7.387969000	7.684144000	-1.603629000
H	-7.661956000	8.879803000	-2.876471000
H	-9.303491000	8.659646000	-0.223079000
H	-9.601284000	9.656726000	-1.653726000
H	-9.470320000	10.409399000	-0.058184000

E_{tot}=-6418.125671 a.u.

b) Geometry parameters (Å) and E_{tot} (a.u.) of **H_3Hhp** optimized at DFT/CAM-B3LYP/6-31G(d,p) method of D_{3h} symmetry.

C	-1.194031000	3.855812000	0.000000000
C	1.194031000	3.855812000	0.000000000
N	-0.677229000	2.653570000	0.000000000
N	0.677229000	2.653570000	0.000000000
S	0.000000000	5.123887000	0.000000000
N	-2.518818000	4.199969000	0.000000000
N	2.518818000	4.199969000	0.000000000
H	-2.457867000	1.419050000	0.000000000
C	-6.961197000	2.380995000	0.000000000
C	-7.633286000	3.600421000	0.000000000
C	-5.577908000	2.417120000	0.000000000
H	-8.718080000	3.613659000	0.000000000
C	-6.934699000	4.810409000	0.000000000
C	-4.882241000	3.622050000	0.000000000
H	-7.488561000	5.743249000	0.000000000
C	-5.542600000	4.838076000	0.000000000
H	-4.987078000	5.768843000	0.000000000
H	-7.489504000	1.434515000	0.000000000
C	-3.443508000	3.308962000	0.000000000
N	-3.342132000	1.929581000	0.000000000

C	-4.587399000	1.327685000	0.000000000
C	-2.742216000	-2.961967000	0.000000000
C	-3.936247000	-0.893845000	0.000000000
N	-1.959444000	-1.913282000	0.000000000
N	-2.636673000	-0.740287000	0.000000000
S	-4.437417000	-2.561944000	0.000000000
N	-2.377871000	-4.281345000	0.000000000
N	-4.896689000	0.081376000	0.000000000
H	0.000000000	-2.838101000	0.000000000
C	1.418596000	-7.219071000	0.000000000
C	0.698587000	-8.410830000	0.000000000
C	0.695667000	-6.039170000	0.000000000
H	1.229520000	-9.356909000	0.000000000
C	-0.698587000	-8.410830000	0.000000000
C	-0.695667000	-6.039170000	0.000000000
H	-1.229520000	-9.356909000	0.000000000
C	-1.418596000	-7.219071000	0.000000000
H	-2.502426000	-7.203358000	0.000000000
H	2.502426000	-7.203358000	0.000000000
C	-1.143891000	-4.636647000	0.000000000
N	0.000000000	-3.859162000	0.000000000
C	1.143891000	-4.636647000	0.000000000
C	3.936247000	-0.893845000	0.000000000
C	2.742216000	-2.961967000	0.000000000
N	2.636673000	-0.740287000	0.000000000
N	1.959444000	-1.913282000	0.000000000
S	4.437417000	-2.561944000	0.000000000
N	4.896689000	0.081376000	0.000000000
N	2.377871000	-4.281345000	0.000000000
H	2.457867000	1.419050000	0.000000000
C	5.542600000	4.838076000	0.000000000
C	6.934699000	4.810409000	0.000000000
C	4.882241000	3.622050000	0.000000000
H	7.488561000	5.743249000	0.000000000
C	7.633286000	3.600421000	0.000000000
C	5.577908000	2.417120000	0.000000000
H	8.718080000	3.613659000	0.000000000
C	6.961197000	2.380995000	0.000000000
H	7.489504000	1.434515000	0.000000000
H	4.987078000	5.768843000	0.000000000
C	4.587399000	1.327685000	0.000000000
N	3.342132000	1.929581000	0.000000000
C	3.443508000	3.308962000	0.000000000

E_{tot}=-3167.103429 a.u.
